# Noscapine and Apoptosis in Breast and Other Cancers

**DOI:** 10.3390/ijms25063536

**Published:** 2024-03-21

**Authors:** Gloria M. Calaf, Leodan A. Crispin, Edwin O. Quisbert-Valenzuela

**Affiliations:** Instituto de Alta Investigación, Universidad de Tarapacá, Arica 1000000, Chile; kcrispi@gestion.uta.cl (L.A.C.);

**Keywords:** noscapine, apoptosis, Bcl-2, Bax, caspase-8, caspase-9, breast cancer

## Abstract

Breast cancer is the second leading contributor to the age-standardized mortality rate, for both sexes and all ages worldwide. In Europe and the United States, it is the second leading cause of mortality, with an incidence rate of about 2.6 million cases per year. Noscapine, a well-known alkaloid used as a cough suppressant, demonstrated anti-tumor effects by triggering apoptosis in various cancer cell lines and has the potential to become another ally against breast, ovarian, colon, and gastric cancer, among other types of malignancy. Apoptosis plays a crucial role in the treatment of cancer. Noscapine affected *BAX*, *CASP8*, *CASP9*, *NFKBIA*, and *RELA* gene and protein expression in the MCF-7 and MDA-MB-231 cell lines. Gene expression was higher in tumor than in normal tissue, including the BAX expression levels in lung, ovary, endometrium, colon, stomach, and glioblastoma patients; BCL2L1 expression in endometrium, colon, and stomach patients; CASP8 gene expression levels in lung, endometrium, colon, stomach, and glioblastoma patients; RELA in colon, stomach, and glioblastoma patients; and NFKBIA in glioblastoma patients. It can be concluded that noscapine affected genes and proteins related to apoptosis in cancer cell lines and several types of cancer patients.

## 1. Introduction

Breast cancer is the second leading contributor to the age-standardized mortality rate, for both sexes and all ages worldwide, and over 680 thousand deaths occurred due to this disease in 2020 [1]. In Europe and the United States, it is the second leading cause of mortality, with an incidence rate of about 2.6 million cases per year [2]. There are numerous risk factors known to contribute to the development of cancer, such as age, geographic area, and race [2,3]; other known or suspected risk factors for cancer are alcohol, chronic inflammation, hormones, radiation, sunlight, cancer-causing substances, infectious agents, obesity, tobacco, etc. Some of these risk factors can be avoided; however, aging cannot [4].

Apoptosis is a highly regulated cellular process that takes place in both normal and abnormal conditions. It follows distinct biochemical and genetic pathways and is considered a programmed form of cell death [5]. One of the major pathways that induces apoptosis is the mitochondria-dependent pathway. Studies have shown that the Bcl-2 family of proteins, located at the outer membranes of mitochondria, plays a crucial role in regulating this intrinsic pathway. These proteins can either inhibit or promote changes in mitochondrial membrane permeability, which are necessary for the release of cytochrome c and other proteins involved in apoptosis. The released proteins then form apoptosomes, which activate caspases, leading to cell death [6,7,8,9].

Apoptosis, the process of cell death, is initiated by caspases, a type of protease that specifically target cysteine aspartyl residues. These caspases cleave various essential cellular proteins, disrupting the nuclear scaffold and cytoskeleton necessary for normal cell function [5,8]. Tumor cells can develop resistance to apoptosis through the expression of anti-apoptotic proteins like Bcl-2 or by reducing or mutating pro-apoptotic proteins such as Bax [8,10]. It seems that the regulation of both Bcl-2 and Bax expression is controlled by the p53 tumor suppressor gene, since this gene can either suppress or promote changes in mitochondrial membrane permeability, which is required for the release of cytochrome c and other apoptogenic proteins [7,8].

It has been demonstrated that the cysteine-aspartate-specific protease caspase-8 is involved in several cellular processes, including necroptosis, autophagy and pyroptosis, anoikis, cell apoptosis, and T cell differentiation [11]. Enzymes called proteases, which control programmed cell death, are members of the caspase protein family. Caspase-9 is an essential component of the mitochondrial apoptotic pathway and a member of the intrinsic pathway. When cells are under stress, the release of cytochrome c from mitochondria starts the intrinsic route. Cytochrome c forms a multiprotein complex known as an apoptosome through interactions with pro-caspase-9, apoptotic protease activating factor 1, and deoxyadenosine triphosphate. The apoptosome activates caspase-9, which triggers a cascade of effector caspases [12].

Apoptosis is regulated by the Bcl-2 family, which includes the anti-apoptotic and pro-apoptotic proteins (Bax, Bok, Bak, etc.). These proteins often interact in dimers and act as apoptotic switches. Anti-apoptotic proteins, such as Bcl-2, block the functions of these pro-apoptotic proteins. The pro-apoptotic and anti-apoptotic protein–protein interactions must be inhibited to prevent tumor cells from escaping apoptosis [13]. Bcl-2 family proteins are tightly involved in the regulation of intrinsic apoptosis [14].

NFκB, a transcription factor, plays a crucial role in gene expression related to cell survival. It promotes the upregulation of anti-apoptotic and pro-survival genes, including those from the Bcl-2 family and IAP proteins. In breast cancer cell lines, NFκB increased the expression of anti-apoptotic genes and proteins while decreasing pro-apoptotic ones [15,16,17]. The translocation of NFκB to the nucleus is preceded by phosphorylation, ubiquitination, and the proteolytic degradation of IκBα, an inhibitor of NFκB [15,18,19,20]. Previous studies have demonstrated higher levels of NFκB polypeptides (both p100 and p52) in mammary carcinoma cell lines and primary tumors compared to normal breast cells [21,22,23].

A single genetic change will rarely lead to the development of a malignant tumor [24]. If the cancer cells can evade apoptotic stimuli, they can survive and acquire drug resistance [25]. The tumors may achieve similar ends by increasing the expression of anti-apoptotic regulators (Bcl-2, Bcl-xL) or of survival signals (Igf1/2), by downregulating pro-apoptotic factors (Bax, Bim, Puma), or by short-circuiting the extrinsic ligand-induced death pathway [26].

This review analyzed the role of noscapine and its anti-tumor activity demonstrated by triggering apoptosis on various cancer cell lines from different tissues.

## 2. Noscapine

Noscapine, a phthalide isoquinoline alkaloid derived from opium obtained from *Papaverum somniferum*, has been extensively researched and used as an oral anti-tussive agent [27,28,29]. It has demonstrated anti-tumor properties against various cancer types, including lung, cervical, prostate, ovarian, and breast cancer, both in vitro and in vivo, while exerting minimal adverse side effects [30,31,32,33,34].

For a long time, noscapine has been used as an effective oral medication to treat cough, and it has been recognized as a highly beneficial drug with minimal side effects. Some of its valuable advantages over other microtubule drugs are its low toxicity, water solubility, and suitability for oral administration [28,30,35,36]. When taken orally, noscapine has demonstrated a significant decrease in tumor size, while also showing minimal to no toxic effects on the body [37,38].

Noscapine has demonstrated minimal or negligible toxicity towards various organs, including the kidney, heart, liver, bone marrow, spleen, and small intestine. Furthermore, it does not inhibit primary humoral immune responses in mice. In addition to these benefits, noscapine exhibits good oral tolerance and has a low risk of addiction [35,39,40]. Other advantages of noscapine are its water solubility and suitability for oral administration, making it superior to many other anti-microtubule drugs [41]. The present review is focused on showing that noscapine has anti-tumor activity in different cell lines, as well as its molecular mechanism of action and its comparison with other drugs.

A sub-therapeutic dose of noscapine (300 mg/kg/day) was administered orally to nude mice with implanted tumors [31]. The results revealed that various organs, including the liver, kidney, spleen, lung, heart, brain, gut, and sciatic nerve, showed no significant differences or pathological abnormalities when compared to the control group [31].

One group of drugs that target microtubules, such as colchicine, nocodazole, and the vinca alkaloids, inhibit the formation of microtubules. Another group, including toxoids and epothilones, promotes the formation of microtubules and stabilizes them. However, these drugs disrupt the normal dynamics of microtubules, leading to cell cycle arrest, usually during prometaphase, blocking the progression of mitosis and ultimately causing cell death [30,31,34,42]. Although microtubule-targeting drugs like vinca alkaloids and taxanes have been proven to be effective in treating different types of cancer in humans, their clinical success has been limited due to the development of drug resistance and the associated toxicities, such as leukocytopenia, diarrhea, alopecia, and peripheral neuropathies caused by the blockage of axonal transport [41,43].

Furthermore, the discovery of new tubulin ligands with antimitotic properties and the identification of their binding sites and modes of action hold promise regarding the developing a new generation of structure-based drugs with improved potential to treat cancer [44,45,46]. Microtubules, which consist of repeating α/β-tubulin heterodimers, are crucial cytoskeletal polymers found in all eukaryotes; these highly dynamic polymers, composed of tubulin subunits, play a vital role in various cellular processes, including cell division, cell motility, and cytoplasmic organization, both in vivo and in vitro [44,45,46].

The dynamic instability of microtubules is driven by the binding and hydrolysis of GTP by tubulin subunits [44,45,46]. Each tubulin monomer binds to one GTP molecule. The binding to α-tubulin at the N-site is permanent, while the binding to β-tubulin at the E-site is replaceable. Polymerization can only occur with dimers containing GTP in their E-site, but, once polymerized, this nucleotide is hydrolyzed and becomes permanent [44,45,46]. The GTP cap model is the most widely accepted hypothesis to explain dynamic instability. According to this model, the microtubule structure is supported by a layer of tubulin subunits at the ends that still contain GTP, while the body of the microtubule consists of GDP–tubulin subunits. When this cap is randomly lost, the microtubule undergoes rapid depolymerization. The assembly and stability of microtubules are regulated by the nucleotide state of tubulin and can be influenced by cellular factors that either stabilize or destabilize microtubules at different locations in the cell or stages in the cell cycle. Disruption of microtubule dynamics can result in the formation of abnormally stable or unstable microtubules, which hinders the normal rearrangement needed for cell division [39,44,45].

Various anti-tubulin agents have been categorized into three main groups based on their binding sites: the colchicine-binding site, those binding at the vinblastine site, and the taxol-binding site. Functionally, these antimitotic ligands can be divided into two classes: those that inhibit microtubule assembly and those that promote microtubule assembly and stabilization. However, regardless of their differences, these agents primarily induce mitotic arrest by inhibiting normal dynamic instability at low concentrations [44].

As described in [47], noscapine has been tested in phase I and II clinical trials for various human cancers, although its exact mechanism of action as a stabilizer or destabilizer of microtubules has not been determined yet. The drug demonstrated the ability to change the dynamics of microtubule assembly, leading to cell cycle arrest during mitosis and apoptosis in multiple mammalian tumor cell lines [45,48]. However, unlike other microtubule-targeting anti-cancer drugs, noscapine did not have an impact on microtubule polymerization or the overall polymer mass of tubulin, even at high concentrations. Furthermore, when applied to tissue culture cells, noscapine did not cause significant deformation in cellular microtubules. Instead, it specifically altered the steady-state dynamics of microtubule assembly, which effectively halted the progression of mitosis. This unique characteristic of noscapine suggests that it did not interfere with other microtubule-dependent cellular processes such as organelle distribution and axonal transport, which has been a major concern with many other microtubule-targeting anti-cancer drugs [30,39,49].

A study found that noscapine did not bind to the same site on tubulin as paclitaxel [30]. Additionally, fluorescence experiments showed that noscapine did not compete with colchicine [30,38,50]. Nonetheless, an in silico investigation revealed a potential binding site for noscapine at the α/β-tubulin interface near the colchicine-binding site. This finding was supported by a study that employed molecular docking and molecular dynamic simulations to identify the predicted binding site of noscapine at the intradomain region of α- and β-tubulin [38]. Upon the binding of noscapine, there was an observed increase in the stability of the tubulin elements at the E-site components and a decrease in the dynamical motions of certain parts of tubulin located alongside the protofilament. These effects interfered with the longitudinal interactions in microtubules, suggesting a positive impact on microtubule polymerization [38,51].

### Analogs of Noscapine

**Figure 1:**
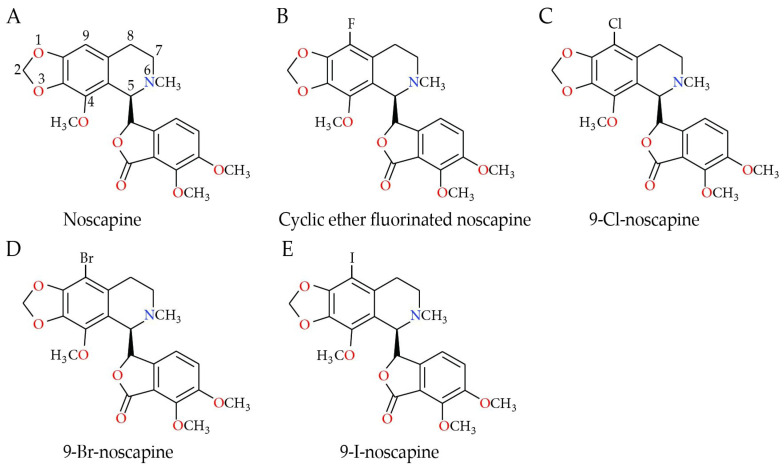
Structures of (**A**) noscapine, (**B**) cyclic ether fluorinated noscapine analog (CEFNA), (**C**) 9-Cl-noscapine (EM015), (**D**) 9-Br-noscapine, and (**E**) 9-I-noscapine.

Analogs of noscapine have been extensively studied. A recent study [31] reported compounds derived from noscapine, known as noscapine analogs (Figure 1A–E), which show great potential in cancer treatment.

Among these analogs, the cyclic ether fluorinated noscapine analog (CEFNA) has demonstrated anti-cancer properties [52]. It has been observed that CEFNA inhibits the growth of, specifically, the MCF-7 and MDA-MB-231 breast cancer cell lines. When these cells were treated with CEFNA at various doses (5, 10, and 25 µM), it caused the formation of multipolar spindles and condensed chromosomes, indicating a halt in the cell cycle at the G2/M phase. Moreover, CEFNA-treated MCF-7 cells exhibited apoptosis, as evidenced by the presence of fragmented micronuclei and apoptotic bodies after 72 h of treatment with 25 µM CEFNA [52].

Another analog, 9-Cl-noscapine (EM015), stood out as it contained a chlorine atom in position 9 of the isoquinoline ring system (9-Cl-noscapine). Compared to noscapine, 9-Cl-noscapine exhibited higher affinity for binding to tubulin, resulting in the inhibition of cellular proliferation in various breast cancer cell lines, including MCF-7, MDA-MB-231, ER-MDA-MB-231, BT474, SK-Br3, and T47D. The IC_50_ value of 9-Cl-noscapine ranged from 2 to 10 µM, which was 15- to 20-fold lower than that of noscapine. Moreover, 9-Cl-noscapine, similar to noscapine, disrupted the cell cycle of breast cancer cells by inducing spindle abnormalities, leading to apoptosis specifically in the G2/M cell cycle phase. Notably, 9-Cl-noscapine required fewer doses than noscapine to achieve these effects [31]. Furthermore, this study demonstrated that 9-Cl-noscapine effectively inhibited the growth of human breast xenografts compared to the noscapine and control groups. It not only prevented tumor growth but also significantly prolonged the survival of mice almost three-fold. Additionally, the use of 9-Cl-noscapine did not lead to any metastatic lesions or disruption of the hydro-electrolytic acid–base balance. The immune system, specifically the B and T cell lineages, remained unaffected, with minimal or no side effects [31].

In a study [34], the compound 9-Br-noscapine was found to inhibit cell growth in the MDA-MB-231 (estrogen, progesterone, and ERB2 receptor negative) cell line. Through Western blot analysis, it was observed that treating MDA-MB-231 cells with 9-Br-noscapine at various concentrations (ranging from 0.01 µM to 1000 µM) led to an increase in Bax protein levels and a decrease in Bcl-2 levels. This resulted in an increase in the Bax/Bcl-2 ratio in a time-dependent manner. Additionally, the activation of caspase-3 and PARP cleavage, along with mitochondrial damage and cytochrome c release, induced apoptosis in MDA-MB-231 cells [34]. The oral administration of 9-Br-noscapine to mice with implanted tumors derived from MDA-MB-231 cells resulted in a reduction in tumor volume on days 16, 22, and 30. The tumor volume reduction was 45%, 59%, and 74%, respectively, compared to mice that received only the vehicle solution. Immunohistochemical analysis revealed the widespread expression of cleaved caspase-3, cleaved PARP, and TUNEL-positive cells in the remaining small regressed tumor sections of the 9-Br-noscapine treatment groups. In conclusion, the study demonstrated that the regression of tumor xenografts was a result of apoptosis induced by 9-Br-noscapine [34].

Furthermore, 9-Br-noscapine was administered to nude mice with implanted tumors derived from MDA-MB-231 cells. Tissue sections of the liver, kidney, spleen, brain, heart, lung, gut, and sciatic nerve were examined using H&E staining. The results indicated that 9-Br-noscapine did not cause any detectable pathological abnormalities or metastatic lesions in these organs. Furthermore, a complete blood count analysis revealed that 9-Br-noscapine treatment did not alter the counts of red blood cells or white blood cells, hemoglobin concentrations, or hematocrit in mice with hormone-refractory xenograft tumors [34].

Halogenated noscapine analogs (9-F-noscapine, 9-Cl-noscapine, 9-Br-noscapine, and 9-I-noscapine) have greater tubulin-binding activity compared to noscapine. These medications have a more pronounced impact on the cell cycle profile, causing heightened arrest at the G2/M phase when compared to noscapine. At concentrations of 5 and 10 µM, the effects of these halogenated compounds on the cell cycle differ in terms of the extent of their detrimental effects, increasing the percentage of sub-G1 cells with hypodiploid DNA content, which is indicative of apoptosis [34,43]. The authors discovered that when the MCF-7 (estrogen and progesterone receptor positive, ErbB2 receptor negative) cell line was treated with halogenated noscapine, there was a noticeable presence of spindles and condensed chromosomes that were not properly organized at the metaphase plate, indicating the onset of mitotic arrest as early as 12 h and maximizing at 24 h of drug treatment. These findings demonstrated that the insertion of halogens into noscapine enhanced its tubulin-binding activity and influenced its potential as a therapeutic agent for various types of cancer cells, particularly the MCF-7 cell line [43].

## 3. Noscapine’s Effect in Several Types of Cancer

### 3.1. Breast Cancer

Several studies have demonstrated that noscapine decreased the levels of the NFκB, P-IκBα, Bcl-2, and survivin proteins, all known as anti-apoptotic proteins. On the other hand, the expression of the cleaved IκBα, PARP, Bax, caspase-8, caspase-9, and caspase-3 proteins, which are pro-apoptotic proteins, were increased compared to the control group. The ratio of Bax/Bcl2 observed in the control group was lower than that observed when treated with noscapine [34,37,53].

A previous study [54] demonstrated that noscapine, a natural opium alkaloid with purity of 97%, induced apoptosis in breast cancer cell lines, specifically MDA-MB-231 and MCF-7. This study compared the effects of noscapine on MCF-10F, a normal breast cell line, used as a control. Such results showed that noscapine exhibited lower toxicity in normal cells while effectively functioning as an anti-cancer agent by triggering apoptosis in breast cancer cells. This was supported by the findings of increased Bax gene and protein expression in all three cell lines, as well as a decrease in the Bcl-xL gene and Bcl-2 protein expression. Noscapine increased Bax protein expression in the MCF-10F cell line, but there was no significant effect on Bcl-2 expression. However, noscapine increased Bax protein expression and decreased Bcl-2 expression in the MCF-7 cell line. Similarly, noscapine increased Bax protein expression and decreased Bcl-2 expression in the MDA-MB-231 breast cancer cell line; there was also an increase in the Bax/Bcl-2 ratio in all three types of cells. The amount of 53 µM noscapine induced an increase in the Bax/Bcl-2 protein expression ratio from 0.03 to 0.70 in the MCF-10F cell line This ratio represents the balance between the pro-apoptotic protein Bax and the anti-apoptotic protein Bcl-2, suggesting a shift towards apoptosis. Similarly, in the MCF-7 cell line treated with 30 µM noscapine, the Bax/Bcl-2 ratio increased from 0.71 to 1.08. Furthermore, treatment with 20 µM noscapine resulted in an increase in the Bax/Bcl-2 ratio from 0.99 to 3.64 in the MDA-MB-231 cell line. Noscapine not only upregulated the expression levels of the caspase-8 and caspase-9 genes but also facilitated the cleavage of caspase-8. Additionally, noscapine downregulated the expression of anti-apoptotic genes and proteins while increasing the expression of pro-apoptotic genes and proteins. These effects may be attributed to the downregulation of the NFκB gene and protein expression. NFκB is a transcription factor associated with breast cancer initiation and progression. Furthermore, noscapine was found to enhance the expression of the IκBα gene, *NFKBIA* [54]. Our previous work [54] also indicated that noscapine-treated MCF-10F cells increased *Bax*, *caspase-8*, and *IκBα* gene expression. However, there was no change in *Bcl-xL*, *caspase-9*, or *NFκB* under the effect of this drug. Noscapine increased *Bax*, *caspase*-*9*, and *IκBα* gene expression while decreasing the levels of *Bcl*-*xL*, and *NFκB* in MCF-7. *Caspase*-*8* showed no significant effect of noscapine. Noscapine-treated MDA-MB-231 cells showed increased *Bax*, *caspase*-*9*, and *Caspase*-*8* gene expression and decreased levels of *Bcl*-*xL*, and *NFκB*. *IκBα* showed no significant effect of noscapine.

Additionally, some authors [54] indicated that noscapine upregulated the caspase-8 and caspase-9 gene expression levels in the MCF-10F and MDA-MB-231 breast cancer cell lines. It also promoted the cleavage of caspase-8, suggesting the involvement of both extrinsic and intrinsic apoptosis pathways in noscapine-induced apoptosis.

On the other hand, noscapine can be used in combination with certain drugs to treat various types of cancer. For example, doxorubicin is commonly used as a chemotherapy agent for patients with metastatic breast cancer [37]. When noscapine and doxorubicin were used together (30 µM and 0.4 µg/mL, respectively), it was observed that the expression of certain proteins involved in apoptosis, such as Bax, caspase-8, caspase-9, caspase-3, and cleaved caspase-3, increased. Additionally, the expression of NFκB, IκBα, P-IκBα, and Bcl-2 decreased when compared to the control group. When noscapine and doxorubicin were used in combination, the expression of the VEGF protein decreased compared to each substance alone, and there was a decrease in survivin protein expression compared to the single-drug treatment and the control group [37]. Furthermore, the combined treatment induced apoptosis in 65% of the tumor cells, whereas noscapine and doxorubicin alone induced apoptosis in 20% and 32% of the tumor cells, respectively [37].

Noscapine, either alone or in combination with doxorubicin, demonstrated efficacy against triple-negative breast cancer cell lines and enhanced the anti-cancer effects of doxorubicin synergistically [37]. This effect was achieved by deactivating the NFκB and anti-angiogenic pathways and promoting apoptosis. As a result, these findings suggest that a combination of orally administered noscapine and doxorubicin could be a potential therapy for aggressive triple-negative breast cancer.

A previous study indicated that the oral administration of noscapine at doses ranging from 150 to 550 mg/kg/day resulted in a significant reduction in tumor volume in MDA-MB-231 xenografts. However, the combined treatment proved to be the most effective in inhibiting tumor growth compared to individual treatments with either doxorubicin or noscapine [37].

Cell proliferation was evaluated by observing the effects of docetaxel, tamoxifen, and noscapine in the MCF-7 and MDA-MB-231 cell lines; although noscapine showed cytotoxic effects in a time- and dose-dependent manner, MDA-MB-231 cells were more susceptible to its effects; however, noscapine inhibited MCF-7 and MDA-MB-231 cells’ proliferation in vitro, which was comparable to the effects of tamoxifen and docetaxel [55]. The combination of N-3-Br-benzyl-noscapine (Br-Bn-Nos), a derivative of noscapine, and docetaxel was demonstrated to have improved anti-cancer potential compared to the single regimen [56]. In drug-resistant xenografts, noscapine at low concentrations with docetaxel decreased the tumor volume in comparison with each substance alone and downregulated the expression of anti-apoptotic factors and multidrug resistance proteins [57].

### 3.2. Clinical Relevance Analyzed by Bioinformatics in Breast Cancer Patients

Identifying genes linked to specific tissues has proven valuable in elucidating their biological roles and understanding diseases such as breast cancer (BRCA) and its various subtypes, including BRCA-Basal, BRCA-Her2, BRCA-Lum-A, and BRCA-Lum-B [58].

Hence, genes such as *BAX*, the BCL2-associated X gene; *BCL2L1*, the BCL2-like 1 gene (Bcl-xL); *CASP8*, the caspase-8 gene; *CASP9*, the caspase-9 gene; *RELA*, the RELA proto-oncogene (NF-kB subunit); and *NFKBIA*, the NFKB inhibitor alpha gene (IkBα) were extracted from the Tumor Immune Estimation Resource database v 2.0 (TIMER2.0, http://timer.cistrome.org, accessed on 14 September 2023) [59]. The results showed whether such genes could have therapeutic target potential, to discover the co-expression patterns of genes across TCGA cancer types such as BRCA. The ER status raw data were extracted from the University of California, Santa Cruz (https://xena.ucsc.edu, accessed on 14 September 2023) UCSC Xena functional genomics explorer [60].

#### 3.2.1. Gene Correlation in Breast Cancer Patients

**Figure 2:**
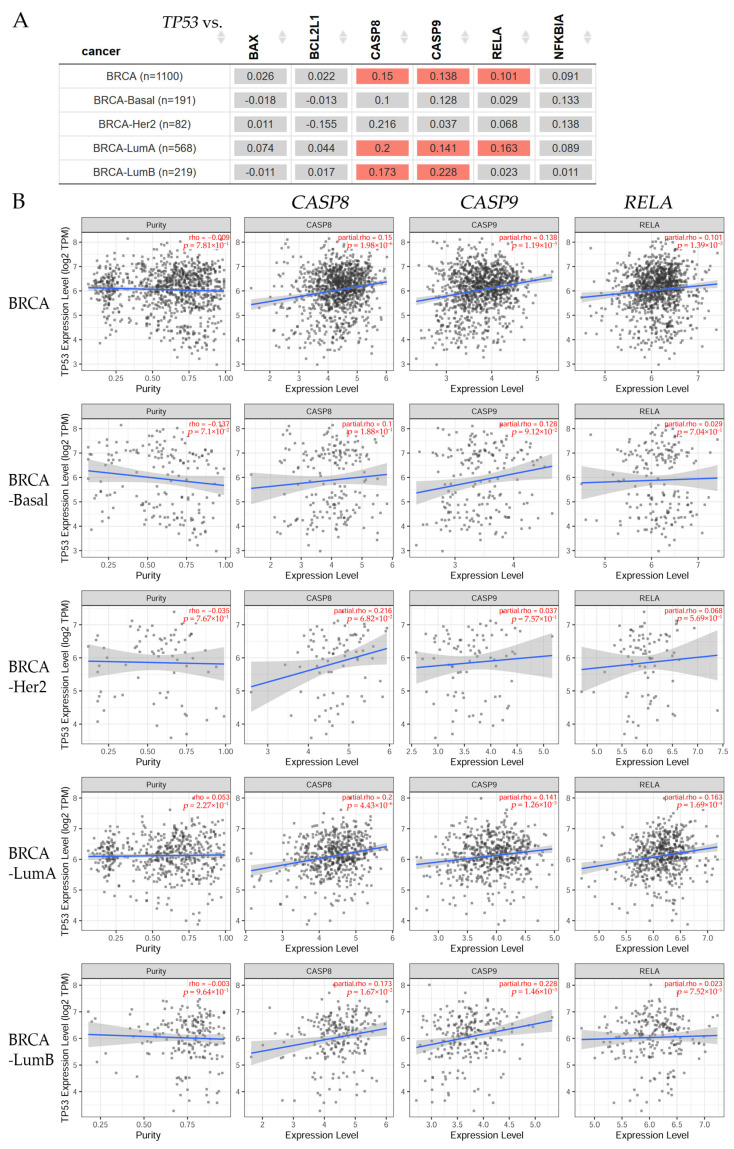
(**A**) The heatmap table shows the correlations between *TP53*, the tumor protein p53 gene, and *BAX*, the BCL2-associated X gene; *BCL2L1*, the BCL2-like 1 gene; *CASP8*, the caspase-8 gene; *CASP9*, the caspase-9 gene; *NFKBIA*, the NFKB inhibitor alpha gene; and *RELA*, the RELA proto-oncogene (NFkB, Rel A) in breast invasive carcinoma (BRCA) subtypes. The red color indicates a statistically significant positive correlation (Spearman’s, *p* < 0.05) and gray denotes a non-significant result. (**B**) Box plots show significant correlations between *TP53* expression with purity adjustment (left) and *CASP8*, *CASP9*, and *RELA* gene expression levels (right) in BRCA subtypes. Correlation values for each analysis are stated in red on the right (Spearman’s, *p* < 0.05). Raw data were extracted from TIMER2.0 (http://timer.cistrome.org), accessed on 14 September 2023 [59].

Correlations were found between TP53 gene expression and *BAX*, *BCL2L1*, *CASP8*, *CASP9*, *NFKBIA*, and *RELA* (Figure 2).

The results in Figure 2A show that there was no correlation between *TP53* and *BAX*, *BCL2L1*, and *NFKBIA* gene expression levels; however, there was a significant (*p* < 0.05) difference between *TP53* gene expression and *CASP8* and *CASP9* for BRCA-LumA and BRCA-LumB patients. The correlation between *TP53* and *RELA* was significant (*p* < 0.05) for BRCA-LumA patients, corroborated by box plots (Figure 2B) showing significant (*p* < 0.05) correlations between *TP53* expression with purity adjustment (left) and *CASP8*, *CASP9*, and *RELA* gene expression levels (right).

#### 3.2.2. Differential Gene Expression Levels between Tumor and Normal Tissues across Various Breast Cancer Subtypes

**Figure 3:**
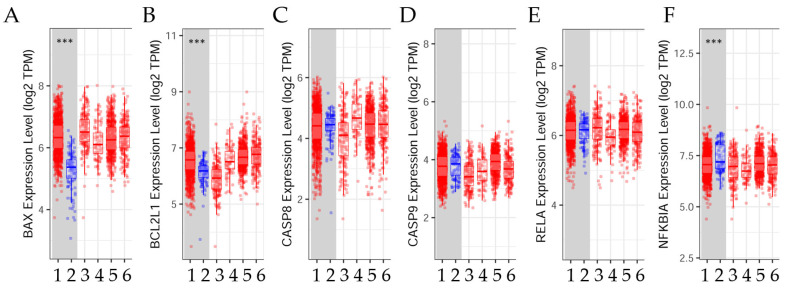
Differential gene expression levels between tumor and normal tissue in breast invasive carcinoma (BRCA). The box plots show the distribution of gene expression levels of (**A**) *BAX*, the BCL2-associated X gene; (**B**) *BCL2L1*, the BCL2-like 1 gene; (**C**) *CASP8*, the caspase-8 gene; (**D**) *CASP9*, the caspase-9 gene; (**E**) *RELA*, the RELA proto-oncogene; and (**F**) *NFKBIA*, the NFKB inhibitor alpha gene in tumor versus normal tissue (Wilcoxon rank-sum test, ***: *p* < 0.001). Raw data were extracted from the Tumor Immune Estimation Resource database v 2.0 (TIMER2.0, http://timer.cistrome.org), accessed on 14 September 2023 [59]. (1) BRCA. Tumor (*n* = 1093). (2) BRCA. Normal (*n* = 112). (3) BRCA-Basal. Tumor (*n* = 190). (4) BRCA-Her2. Tumor (*n* = 82). (5) BRCA-LumA. Tumor (*n* = 564). (6) BRCA-LumB. Tumor (*n* = 217).

Studies analyzed the differential gene expression levels between tumor and normal tissue across various breast cancer subtypes, as shown in Figure 3.

The results show that *BAX* and *BCL2L1* were significantly (*p* < 0.001) higher in the tumor tissue than in the normal tissue when comparisons were made, whereas *NFKBIA* was significantly (*p* < 0.001) higher in the normal than in the cancer tissue. There was no significant relationship between tumor and normal tissue in terms of *CASP8*, *CASP9*, and *RELA* gene expression.

#### 3.2.3. Estrogen Receptor Status

**Figure 4:**
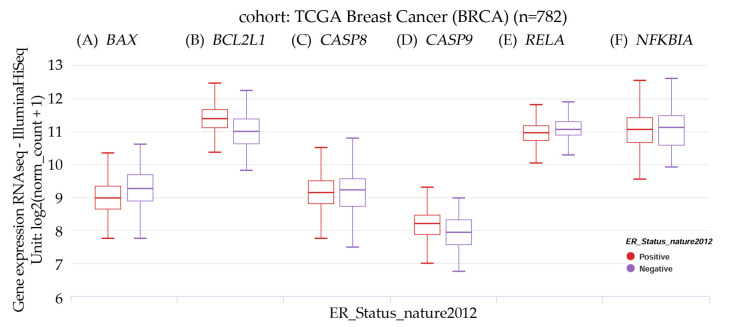
Xena Chart View showing box plot transcript expression of (**A**) *BAX*, the BCL2-associated X gene; (**B**) *BCL2L1*, the BCL2-like 1 gene; (**C**) *CASP8*, the caspase-8 gene; (**D**) *CASP9*, the caspase-9 gene; (**E**) *RELA*, the RELA proto-oncogene; and (**F**) *NFKBIA*, the NFKB inhibitor alpha gene in breast invasive carcinoma (BRCA). Cohort: TCGA Breast Cancer (BRCA), *n* = 782, stratified by nature2012 for estrogen receptor status (ER) (one-way ANOVA, *p* < 0.05). Raw data were extracted from the University of California, Santa Cruz (ena.ucsc.edu) UCSC Xena functional genomics explorer, accessed on 14 September 2023 [60].

Bioinformatic studies analyzed *BAX*, *BCL2L1*, *CASP8*, *CASP 9*, *RELA*, and *NFKBIA*, gene expression, and estrogen receptor status, as seen in Figure 4.

The results indicated that those BRCA patients characterized by *BAX* and *RELA* gene expression had a significant (*p* = 1.097 × 10^−8^ and *p* = 0.0001753, respectively) negative ER status, whereas those with *BCL2L1* and *CASP9* had a significant (*p* = 0.000 and *p* = 6.778 × 10^−7^, respectively) positive ER status. There was no significant difference in those patients having *CASP8* and *NFKBIA* gene expression.

#### 3.2.4. Disease Stage Factor in Several Breast Cancer Subtypes

Gene expression concerning disease stage factors across various breast cancer subtypes is analyzed in Table 1. The clinical relevance of the gene expression associated with the disease stage is important.

The analysis of the clinical stages of patients with breast invasive carcinoma indicated that the *BAX*, *BCL2L1*, *CASP8*, *CASP9*, *RELA*, and *NFKBIA* gene expression levels were significantly (*p* < 0.001) higher in stages 3 and 4 for all BRCA patients, and in stage 4 for BRCA-LumA patients, than in other clinical stages. Additionally, stage 4 showed a significant (either *p* < 0.05 or *p* < 0.01) difference in BRCA-Her2 and BRCA-LumB patients. These genes showed no significance at any stage in BRCA-Basal patients.

### 3.3. Lung Cancer

Gemcitabine, a pyrimidine nucleoside–antimetabolite agent, has shown effectiveness against various types of cancer in humans. When combined with noscapine, the anti-cancer activity of gemcitabine against non-small-cell lung cancer increased in an additive to synergistic manner. This synergism resulted in higher levels of apoptosis compared to treatment with either drug alone. Additionally, in mice with implanted tumors, the combination of noscapine and gemcitabine led to a reduction in tumor volume. This reduction decreased the expression of anti-apoptotic and angiogenic proteins, as well as increasing the expression of pro-apoptotic proteins, within the tumor tissue [37,61].

### 3.4. Ovarian Cancer

Apoptosis was observed in human ovarian carcinoma cells when exposed to noscapine (97% purity) at a concentration of 20 µM, with the extent of apoptosis increasing as the duration of exposure to the drug increased. These findings were evaluated using techniques such as TUNEL and Annexin V [30]. Additionally, noscapine at a concentration of 40 µM exhibited cytotoxic effects on paclitaxel-resistant human ovarian carcinoma cell lines [62].

A recent study showed that noscapine-induced apoptosis in ovarian cancer cell lines was associated with the JNK pathway. The study found that treating the cell lines with noscapine led to increased levels of c-Jun protein and the phosphorylation of c-Jun by JNK; this phosphorylation influenced the expression of apoptotic genes and proteins. Like other microtubule drugs, noscapine also inhibited the microtubule dynamics, caused mitotic arrest, induced apoptosis, and exhibited strong anti-tumor activity [30,63,64]. However, the exact molecular mechanisms that underlie the apoptosis and mitotic arrest induced by anti-microtubule agents, as well as the relationship between these two events, remain unclear.

Cisplatin, a primary chemotherapy drug for ovarian cancer, is known for its high toxicity and the development of resistance in cancer cells. In contrast, the use of noscapine enhanced the sensitivity to cisplatin in ovarian cancer cells resistant to drugs [62]. By combining 2.5 µM noscapine with cisplatin at different concentrations (0, 2, 4, and 8 µg/mL), the proliferation of ovarian cisplatin-resistant cancer cells was reduced, the expression levels of genes and anti-apoptotic proteins were decreased, and the expression of genes and pro-apoptotic proteins was increased compared to using either drug alone. Moreover, the combination of cisplatin and noscapine was found to effectively reduce tumor growth in nude mice [62].

### 3.5. Endometrium

The effect of noscapine was examined on the expression of apoptotic genes, growth scores, angiogenesis, and nitric oxide secretion in the eutopic endometrium in endometriosis patients and normal endometrium patients. Results indicated that the expression of apoptotic genes increased, while the levels of Bcl-2 and Sirt1 decreased [64,65].

### 3.6. Colon Cancer

In a recent study, it was observed that the use of noscapine (97% purity) resulted in a reduction in cell proliferation in human colon cancer cells. The effectiveness of noscapine varied based on the dosage (001, 01, 1, 10, 100, or 1000 µM) and duration of treatment (0, 12, 24, 36, 48, or 72 h). After 72 h, the IC_50_ value for noscapine was found to be 75 µM. Additionally, the study revealed that the cells treated with noscapine experienced cell cycle arrest at the G2/M stage, leading to an increase in apoptosis, as confirmed by flow cytometry analysis. Furthermore, it was observed that at 75 µM, noscapine-induced apoptosis resulted in the upregulation of Bax expression and downregulation of Bcl-2 expression. This apoptotic response was accompanied by an increase in the protein expression of caspase-3 and caspase-9, as well as a decrease in survivin expression. These findings suggest that the induction of apoptosis by noscapine occurs through the mitochondrial pathway [66].

Another study showed that the inhibition of p38 mitogen-activated protein kinase (MAPK) increased the sensitivity of the 5-fluorouracil-resistant SW480 human colon cancer cells to noscapine (at a dose of 25 µM). This was achieved by suppressing proliferation, the induction of cell cycle arrest and apoptosis, and the reversal of multidrug resistance in the SW480 cells treated with 20 µg/mL 5-fluorouracil [67].

### 3.7. Stomach Cancer

In a research study, it was found that noscapine exhibited cytotoxic effects on gastric cancer cell lines (BGC823, SGC7901, MGC803, and HGC27). The cytotoxicity was observed in a dose-dependent and time-dependent manner, with varying concentrations of 0, 50, 100, or 150 µM and time intervals of 0, 12, 24, or 36 h. The cytotoxic activity was attributed to apoptosis, as evidenced by chromatin condensation observed through the DAPI method and quantified using flow cytometry. The apoptotic effect was also found to be dependent on the dosage of noscapine. Furthermore, the expression of the Bax and Bcl-2 proteins was examined, revealing an increase in Bax and a decrease in Bcl-2 levels after treatment with noscapine. Additionally, the activity of caspase-9 and caspase-3 was enhanced following noscapine treatment [68].

### 3.8. Neuroblastoma

The drug noscapine was found to effectively inhibit cellular proliferation in various neuroblastoma cell lines (SK-SY5Y, SH-EP1, SK-N-MC, SK-N-AS, LA1-55N, LA1-5S, NB1643, NB1691, SK-N-SH, and IMR32). This inhibition was dose-dependent, with IC_50_ values ranging from 21 to 101 µM for most of these cell lines. Noscapine induced mitotic arrest at the G2/M phase of the cell cycle. Additionally, treatment with noscapine led to the activation of caspase-3 and the cleavage of PARP in the treated cells. When SK-SY5Y and LA1-5S cells were exposed to noscapine, there was a significant reduction in the levels of survivin mRNA and protein as early as 12 h after treatment. Importantly, the ectopic expression of survivin provided significant protection against noscapine-induced cytoplasmic histone-associated apoptotic DNA fragmentation [69].

### 3.9. Clinical Relevance Analyzed by Bioinformatics in Several Types of Cancer

#### 3.9.1. Gene Correlation in Different Cancer Patients

**Figure 5:**
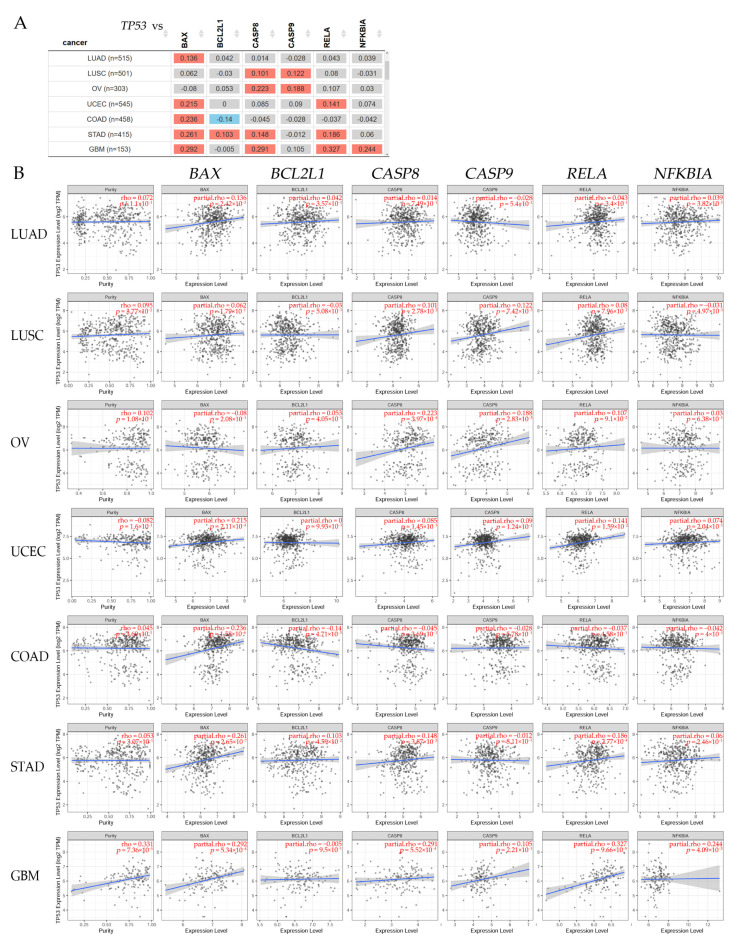
(**A**) Correlations between *TP53*, the tumor protein p53 gene, and *BAX*, the BCL2-associated X gene; *BCL2L1*, the BCL2-like 1 gene; *CASP8*, the caspase-8 gene; *CASP9*, the caspase-9 gene; *NFKBIA*, the NFKB inhibitor alpha gene; and *RELA*, the RELA proto-oncogene (NFkB, Rel A) in different types of cancer. The red color indicates a statistically significant positive correlation (Spearman’s, *p* < 0.05), blue indicates a statistically significant negative correlation (Spearman’s, *p* < 0.05), and gray denotes a non-significant result. (**B**). Box plots show significant correlations between *TP53* expression with purity adjustment (left) and *BAX*, *BCL2L1*, *CASP8*, *CASP9*, *RELA*, and *NFKBIA* gene expression levels (right) in different types of cancer. Correlation values for each analysis are stated in red on the right (Spearman’s, *p* < 0.05). Raw data were extracted from TIMER2.0 (http://timer.cistrome.org), accessed on 10 January 2024 [59]. TCGA abbreviations—LUAD: lung adenocarcinoma; LUSC: lung squamous cell carcinoma; OV: ovarian serous; UCEC: uterine corpus endometrial carcinoma; COAD: colon adenocarcinoma; STAD: stomach adenocarcinoma; GBM: glioblastoma multiforme.

Correlations were found between *TP53* gene expression and *BAX*, *BCL2L1*, *CASP8*, *CASP9*, *RELA*, and *NFKBIA* in lung adenocarcinoma (LUAD), lung squamous cell carcinoma (LUSC), ovarian serous (OV), uterine corpus endometrial carcinoma (UCEC), colon adenocarcinoma (COAD), stomach adenocarcinoma (STAD), and glioblastoma multiforme (GBM), as seen in Figure 5.

The results in Figure 5A indicate that *TP53* had a significantly (*p* < 0.05) positive correlation with *BAX* expression in LUAD, UCEC, COAD, STAD, and GBM patients, being also positively and significantly correlated (*p* < 0.05) with *BCL2L1* expression levels in STAD patients. Meanwhile, it was negatively correlated with *BCL2L1* expression in COAD patients. There was a significant (*p* < 0.05) positive correlation with *CASP8* expression levels in LUSC, OV, STAD, and GBM. The *TP53* and *CASP9* expression levels showed a significant (*p* < 0.05) positive correlation in LUSC and OV patients. *NFKBIA* showed a significant (*p* < 0.05) positive correlation with *TP53* expression in GBM. Additionally, *TP53* presented a significant correlation with the RELA expression levels in COAD, STAD, and GBM patients. Representative box plots (Figure 5B) corroborated these significant (*p* < 0.05) correlations between *TP53* expression with purity adjustment (left) and the *BAX*, *BCL2L1*, *CASP8*, *CASP9*, *RELA*, and *NFKBIA* gene expression levels (right).

#### 3.9.2. Differential Gene Expression Levels between Tumor and Normal Tissue across Different Types of Cancer

**Figure 6:**
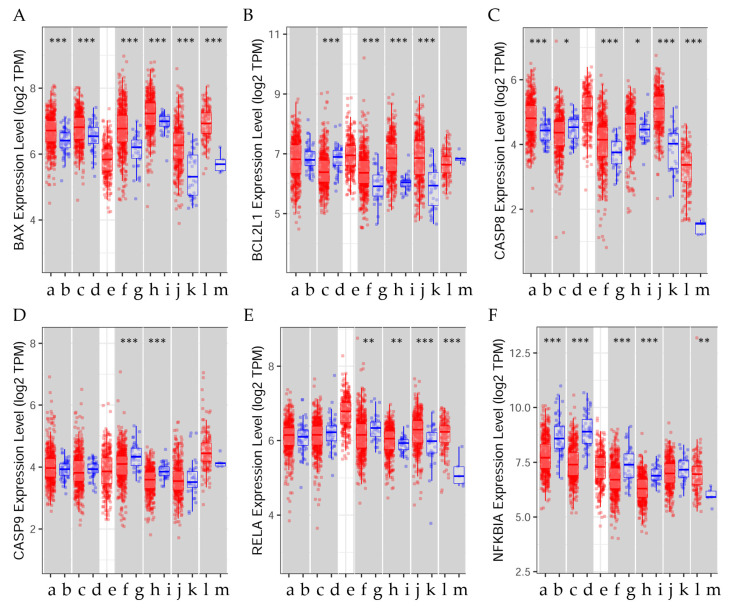
Differential gene expression levels between tumor and normal tissue in different types of cancer. The box plots show the distribution of the gene expression levels of (**A**) *BAX*, the BCL2-associated X gene; (**B**) *BCL2L1*, the BCL2-like 1 gene; (**C**) *CASP8*, the caspase-8 gene; (**D**) *CASP9*, the caspase-9 gene; (**E**) *RELA*, the RELA proto-oncogene; and (**F**) *NFKBIA*, the NFKB inhibitor alpha gene in tumor versus normal tissue (Wilcoxon rank-sum test, *: *p* < 0.05, **: *p* < 0.01, ***: *p* < 0.001). Raw data were extracted from the Tumor Immune Estimation Resource database v 2.0 (TIMER2.0, http://timer.cistrome.org), accessed on 14 September 2023 [59]. (a) LUAD. Tumor (*n* = 515). (b) LUAD. Normal (*n* = 59). (c) LUSC. Tumor (*n* = 501). (d) LUSC. Normal (*n* = 51). (e) OV. Tumor (*n* = 303). (f) UCEC. Tumor (*n* = 545). (g) UCEC. Normal (*n* = 35). (h) COAD. Tumor (*n* = 457). (i) COAD. Normal (n = 41). (j) STAD. Tumor (*n* = 415). (k) STAD. Normal (*n* = 35). (l) GBM. Tumor (*n* = 153). (m) GBM. Normal (*n* = 5).

Studies analyzed the differential gene expression levels between tumor and normal tissue across different types of cancer, as shown in Figure 6.

Figure 6 shows that the *BAX* expression levels were significantly (*p* < 0.001) higher in the tumor tissue than in the normal tissue when comparisons were made in LUAD, LUSC, OV, UCEC, COAD, STAD, and GBM patients. Similarly, *BCL2L1* expression was significantly (*p* < 0.001) higher in the tumor than in the normal tissue in UCEC, COAD, and STAD patients, whereas *CASP8* was significantly (*p* < 0.001) higher in the normal tissue than in the tumor tissue (either *p* < 0.05 or *p* < 0.001) in LUSC. *CASP8* was higher in the tumor tissue than in the normal tissue in LUAD, UCEC, COAD, STAD, and GBM; however, it was significantly (*p* < 0.05) higher in the normal than in the tumor tissue in LUSC patients. The *CASP9* expression level was higher in the normal tissue than the tumor tissue in UCEC and COAD patients. The *RELA* expression level was significantly (*p* < 0.01) higher in the normal tissue than in the tumor tissue in UCEC patients, whereas it was significantly (either *p* < 0.01 or *p* < 0.001) higher in the tumor than the normal tissue in COAD, STAD, and GBM patients. The *NFKBIA* expression level was significantly (*p* < 0.001) higher in the normal than in the cancer tissue in LUAD, LUSC, UCEC, and COAD patients; however, it was significantly (*p* < 0.01) higher in the tumor than the normal tissue in GBM patients.

**Scheme 1:**
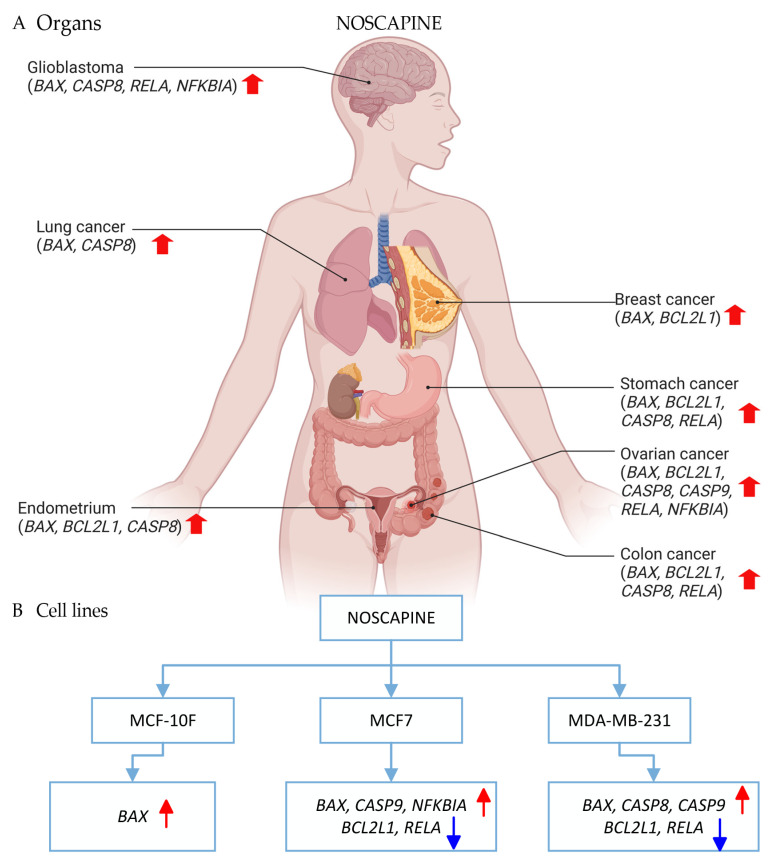
(**A**) Noscapine causes changes in apoptotic genes in cancer in human patients, such as glioblastoma, lung, endometrium, breast, stomach, ovary, and colon. The red arrow indicates an increase and the blue arrow indicates a decrease in gene expression. (**B**) It also causes changes in apoptotic genes and protein expression in breast cancer cell lines.

Scheme 1 shows a comparison between normal and malignant tissue, indicating that the BAX expression levels were higher in the tumor tissue than in the normal tissue in LUAD, LUSC, OV, UCEC, COAD, STAD, and GBM patients. BCL2L1 expression was higher in the tumor than in the normal tissue in UCEC, COAD, and STAD patients, whereas it was higher in the normal tissue than in the tumor tissue in LUSC. The CASP8 gene expression levels were higher in the tumor tissue than in the normal tissue in LUAD, UCEC, COAD, STAD, and GBM; however, it was higher in the normal than in the tumor tissue in LUSC patients. The CASP9 gene expression level was higher in the normal tissue than the tumor tissue in UCEC and COAD patients. The RELA gene expression level was higher in the normal tissue than in the tumor tissue in UCEC patients, whereas it was higher in the tumor than the normal tissue in COAD, STAD, and GBM patients. The NFKBIA gene expression level was higher in the normal than in the cancer tissue in LUAD, LUSC, UCEC, and COAD patients; however, it was higher in the tumor than in the normal tissue in GBM patients. The expression level of NFKBIA was considerably higher in normal tissue than in tumors, suggesting that it may act as a tumor suppressor.

The studies analyzed the effect of noscapine on apoptosis in various cancer cell lines. For example, noscapine increased the gene expression levels of *BAX* in the MCF-10F cell line; it increased *BAX*, *CASP9*, and *NFKBIA* in MCF-7; and it increased *BAX*, *CASP9*, and *CASP8* in the MDA-MB-231 cell line. The results indicated that those BRCA patients characterized by *BAX* and *RELA* gene expression had a negative ER status, whereas those with *BCL2L1* and *CASP9* had a positive ER status. There was no significant difference in those patients having *CASP8* and *NFKBIA* gene expression. The analysis of the clinical stages of patients with breast invasive carcinoma indicated that the *BAX*, *BCL2L1*, *CASP8*, *CASP9*, *RELA*, and *NFKBIA* gene expression levels were higher in stages 3 and 4 for all BRCA patients, and in stage 4 for BRCA-LumA patients, than in other clinical stages. Additionally, stage 4 showed a difference in BRCA-Her2 and BRCA-LumB patients but not at any stage in BRCA-Basal patients.

## 4. Conclusions

It can be concluded that noscapine affected genes and proteins related to apoptosis in cancer cell lines and several types of cancer patients.

## Figures and Tables

**Table 1:** Clinical relevance of genes across various cancer types analyzed by the disease stage factor.

Cancer	BAX	BCL2L1	CASP8	CASP9	RELA	NFKBIA
BRCA (*n* = 1100)	3, 4 ***	3, 4 ***	3, 4 ***	3, 4 ***	3, 4 ***	3, 4 ***
BRCA-Basal (*n* = 191)	N. S.	N. S.	N. S.	N. S.	N. S.	N. S.
BRCA-Her2 (*n* = 82)	4 *	4 *	4 *	4 **	4 *	4 *
BRCA-LumA (*n* = 568)	4 ***	4 ***	4 ***	4 ***	4 ***	4 ***
BRCA-LumB (*n* = 219)	4 **	4 **	4 **	4 **	4 **	4 **

Statistical significance is annotated by the number of stars (Cox proportional hazard model, *: *p* < 0.05; **: *p* < 0.01; ***: *p* < 0.001); 3, 4: clinical stage factor; N. S.: not significant. Data estimated by TIMER2.0 in breast invasive carcinoma, accessed on 14 September 2023 [59].

## Data Availability

TIMER20 is freely available at http://timercistromeorg (accessed on 6 August 2021); the UCSC Xena online exploration tools are freely available at http://xenaucscedu/ (accessed on 20 August 2021). The data generated in the present study may be requested from the corresponding author.
